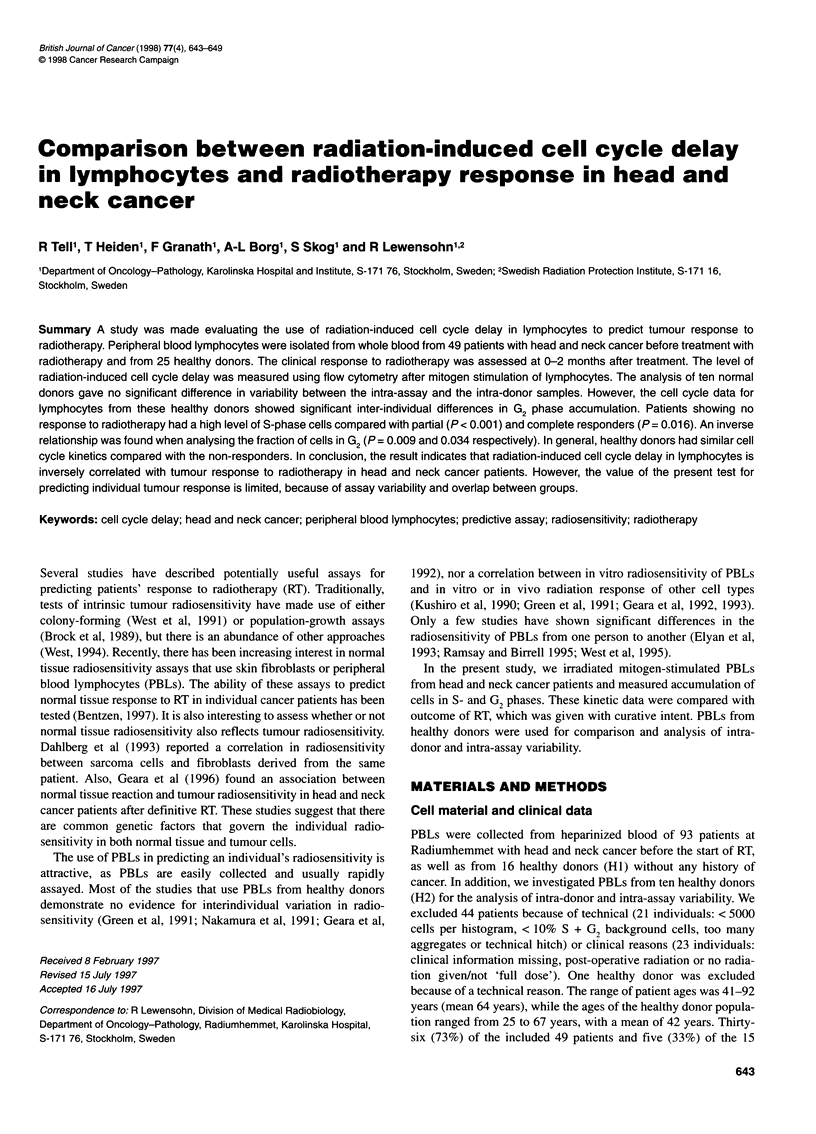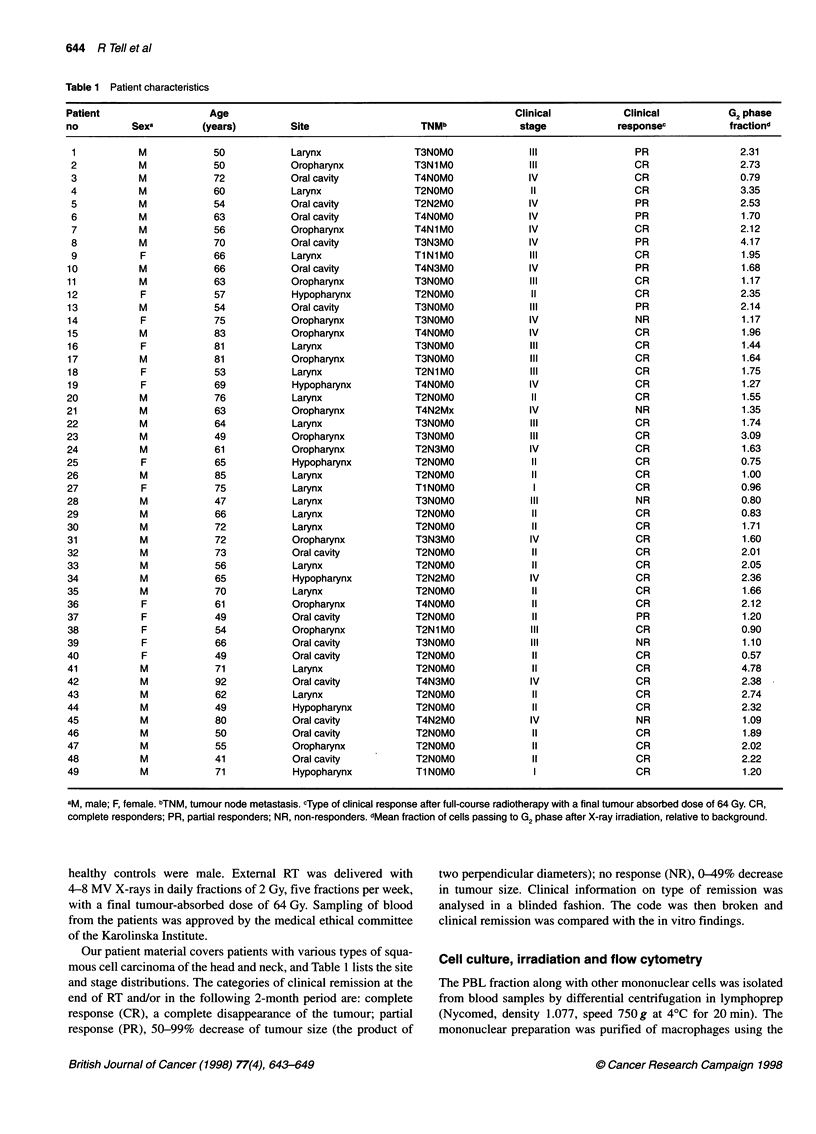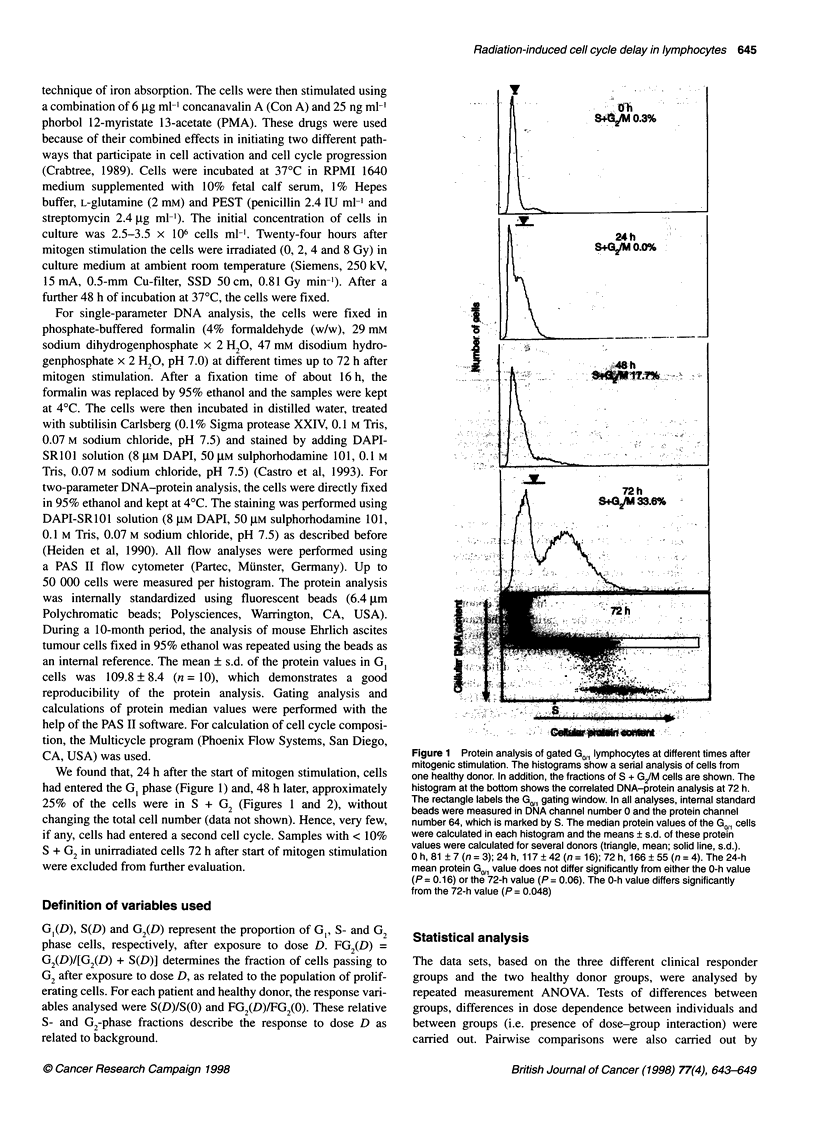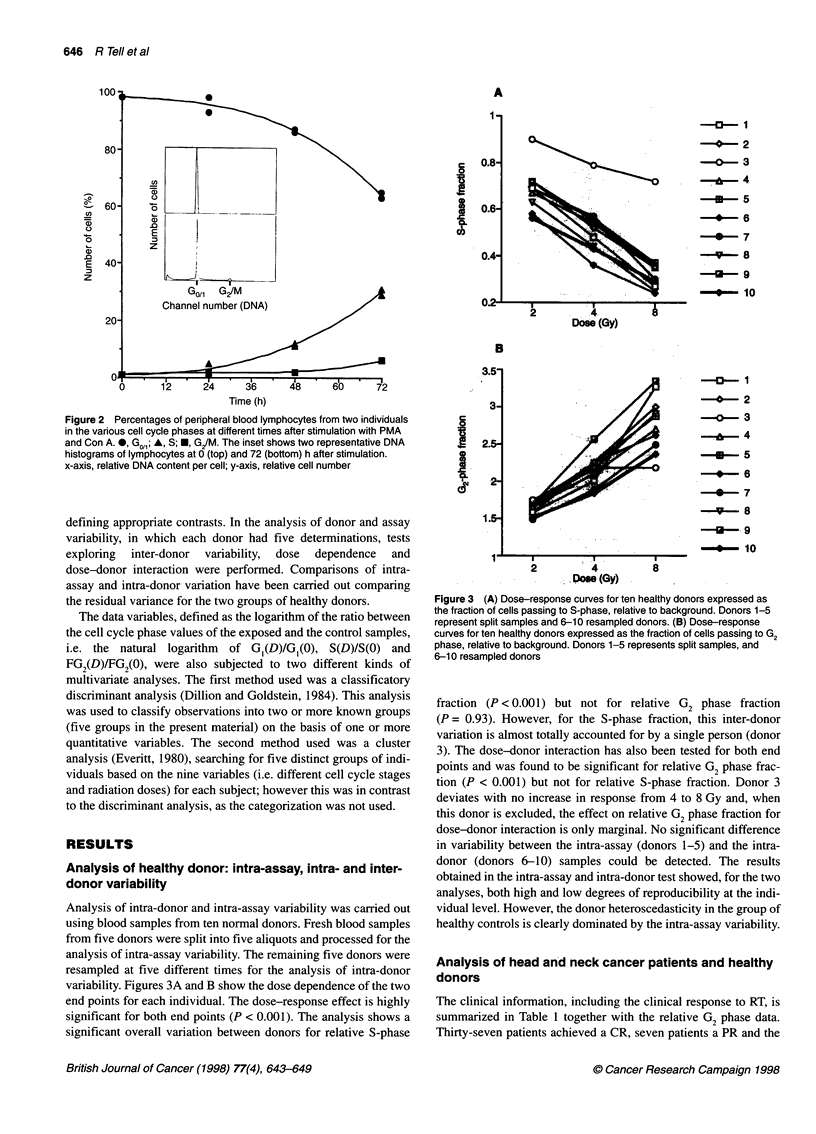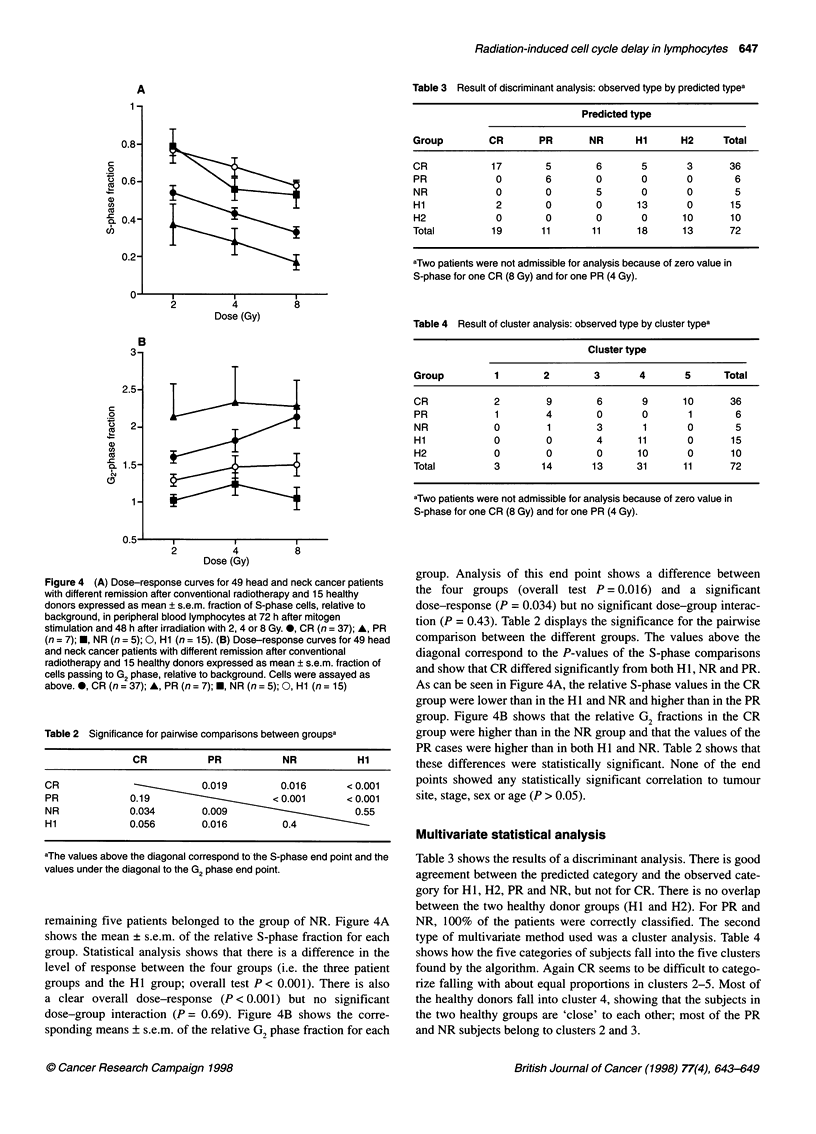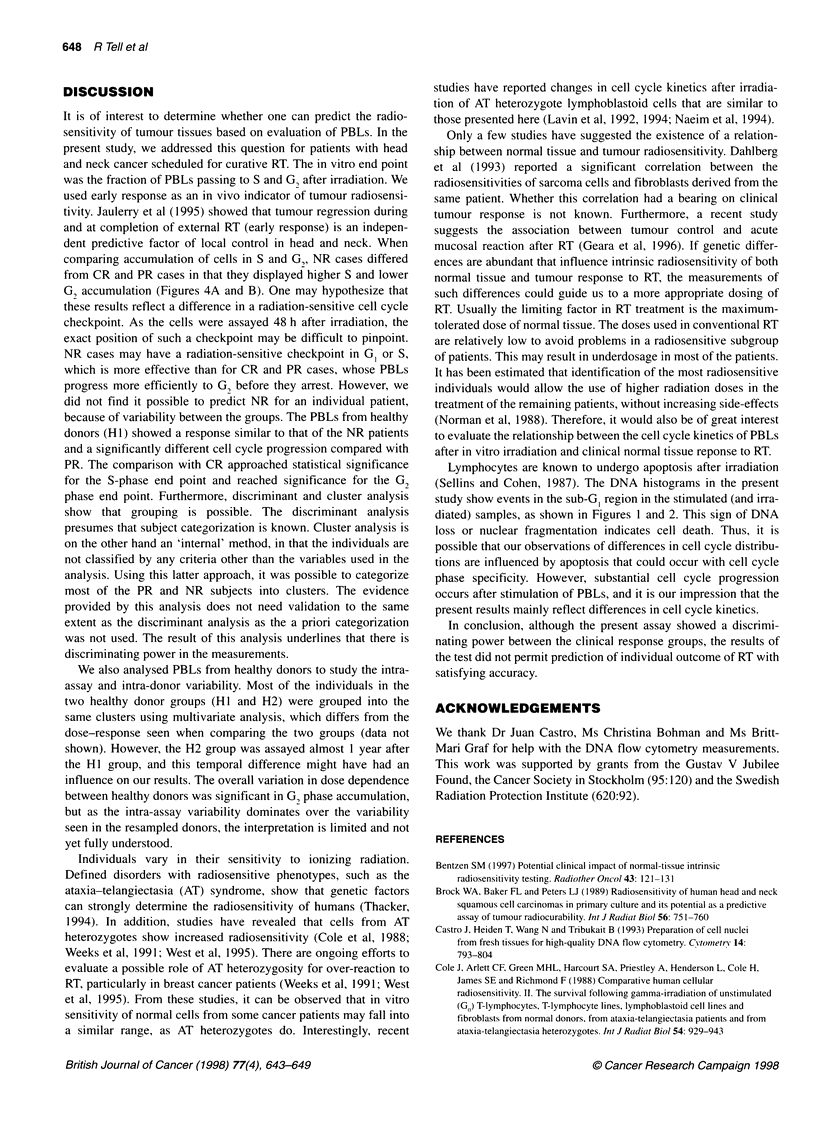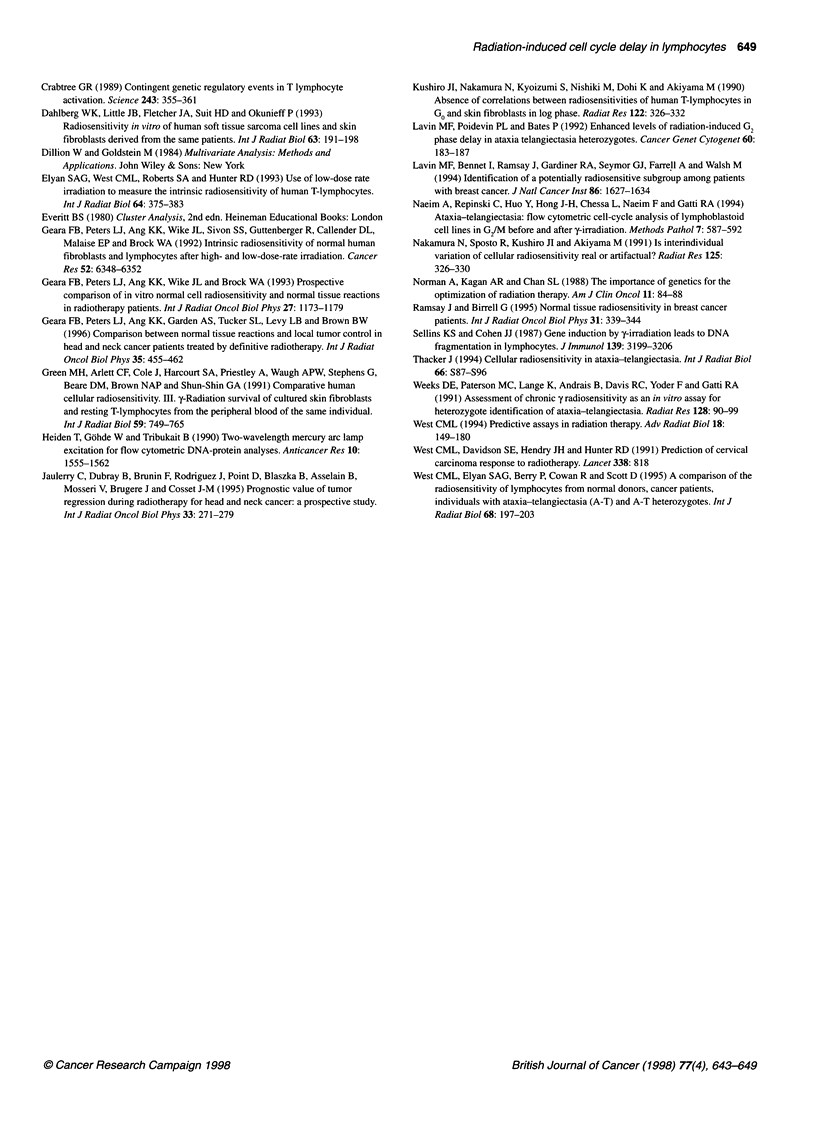# Comparison between radiation-induced cell cycle delay in lymphocytes and radiotherapy response in head and neck cancer.

**DOI:** 10.1038/bjc.1998.103

**Published:** 1998-02

**Authors:** R. Tell, T. Heiden, F. Granath, A. L. Borg, S. Skog, R. Lewensohn

**Affiliations:** Department of Oncology-Pathology, Karolinska Hospital and Institute, Stockholm, Sweden.

## Abstract

A study was made evaluating the use of radiation-induced cell cycle delay in lymphocytes to predict tumour response to radiotherapy. Peripheral blood lymphocytes were isolated from whole blood from 49 patients with head and neck cancer before treatment with radiotherapy and from 25 healthy donors. The clinical response to radiotherapy was assessed at 0-2 months after treatment. The level of radiation-induced cell cycle delay was measured using flow cytometry after mitogen stimulation of lymphocytes. The analysis of ten normal donors gave no significant difference in variability between the intra-assay and the intra-donor samples. However, the cell cycle data for lymphocytes from these healthy donors showed significant inter-individual differences in G2 phase accumulation. Patients showing no response to radiotherapy had a high level of S-phase cells compared with partial (P < 0.001) and complete responders (P = 0.016). An inverse relationship was found when analysing the fraction of cells in G2 (P = 0.009 and 0.034 respectively). In general, healthy donors had similar cell cycle kinetics compared with the non-responders. In conclusion, the result indicates that radiation-induced cell cycle delay in lymphocytes is inversely correlated with tumour response to radiotherapy in head and neck cancer patients. However, the value of the present test for predicting individual tumour response is limited, because of assay variability and overlap between groups.


*British Journal of Cancer* (1998) **77**(4), 643–649
© 1998 Cancer Research Campaign

### Comparison between radiation-induced cell cycle delay in lymphocytes and radiotherapy response in head and neck cancer

**R Tell[1], T Heiden[1], F Granath[1], A-L Borg[1], S Skog[1] and R Lewensohn[1,2]**

[1]Department of Oncology–Pathology, Karolinska Hospital and Institute, S-171 76, Stockholm, Sweden; [2]Swedish Radiation Protection Institute, S-171 16, Stockholm, Sweden

**Summary** A study was made evaluating the use of radiation-induced cell cycle delay in lymphocytes to predict tumour response to radiotherapy. Peripheral blood lymphocytes were isolated from whole blood from 49 patients with head and neck cancer before treatment with radiotherapy and from 25 healthy donors. The clinical response to radiotherapy was assessed at 0–2 months after treatment. The level of radiation-induced cell cycle delay was measured using flow cytometry after mitogen stimulation of lymphocytes. The analysis of ten normal donors gave no significant difference in variability between the intra-assay and the intra-donor samples. However, the cell cycle data for lymphocytes from these healthy donors showed significant inter-individual differences in $G_2$ phase accumulation. Patients showing no response to radiotherapy had a high level of S-phase cells compared with partial ($P < 0.001$) and complete responders ($P = 0.016$). An inverse relationship was found when analysing the fraction of cells in $G_2$ ($P = 0.009$ and 0.034 respectively). In general, healthy donors had similar cell cycle kinetics compared with the non-responders. In conclusion, the result indicates that radiation-induced cell cycle delay in lymphocytes is inversely correlated with tumour response to radiotherapy in head and neck cancer patients. However, the value of the present test for predicting individual tumour response is limited, because of assay variability and overlap between groups.

**Keywords:** cell cycle delay; head and neck cancer; peripheral blood lymphocytes; predictive assay; radiosensitivity; radiotherapy

Several studies have described potentially useful assays for predicting patients' response to radiotherapy (RT). Traditionally, tests of intrinsic tumour radiosensitivity have made use of either colony-forming (West et al, 1991) or population-growth assays (Brock et al, 1989), but there is an abundance of other approaches (West, 1994). Recently, there has been increasing interest in normal tissue radiosensitivity assays that use skin fibroblasts or peripheral blood lymphocytes (PBLs). The ability of these assays to predict normal tissue response to RT in individual cancer patients has been tested (Bentzen, 1997). It is also interesting to assess whether or not normal tissue radiosensitivity also reflects tumour radiosensitivity. Dahlberg et al (1993) reported a correlation in radiosensitivity between sarcoma cells and fibroblasts derived from the same patient. Also, Geara et al (1996) found an association between normal tissue reaction and tumour radiosensitivity in head and neck cancer patients after definitive RT. These studies suggest that there are common genetic factors that govern the individual radiosensitivity in both normal tissue and tumour cells.

The use of PBLs in predicting an individual's radiosensitivity is attractive, as PBLs are easily collected and usually rapidly assayed. Most of the studies that use PBLs from healthy donors demonstrate no evidence for interindividual variation in radiosensitivity (Green et al, 1991; Nakamura et al, 1991; Geara et al, 1992), nor a correlation between in vitro radiosensitivity of PBLs and in vitro or in vivo radiation response of other cell types (Kushiro et al, 1990; Green et al, 1991; Geara et al, 1992, 1993). Only a few studies have shown significant differences in the radiosensitivity of PBLs from one person to another (Elyan et al, 1993; Ramsay and Birrell 1995; West et al, 1995).

In the present study, we irradiated mitogen-stimulated PBLs from head and neck cancer patients and measured accumulation of cells in S- and $G_2$ phases. These kinetic data were compared with outcome of RT, which was given with curative intent. PBLs from healthy donors were used for comparison and analysis of intra-donor and intra-assay variability.

### MATERIALS AND METHODS

### Cell material and clinical data

PBLs were collected from heparinized blood of 93 patients at Radiumhemmet with head and neck cancer before the start of RT, as well as from 16 healthy donors (H1) without any history of cancer. In addition, we investigated PBLs from ten healthy donors (H2) for the analysis of intra-donor and intra-assay variability. We excluded 44 patients because of technical (21 individuals: < 5000 cells per histogram, < 10% S + $G_2$ background cells, too many aggregates or technical hitch) or clinical reasons (23 individuals: clinical information missing, post-operative radiation or no radiation given/not 'full dose'). One healthy donor was excluded because of a technical reason. The range of patient ages was 41–92 years (mean 64 years), while the ages of the healthy donor population ranged from 25 to 67 years, with a mean of 42 years. Thirty-six (73%) of the included 49 patients and five (33%) of the 15

*Received 8 February 1997*
*Revised 15 July 1997*
*Accepted 16 July 1997*

*Correspondence to:* R Lewensohn, Division of Medical Radiobiology, Department of Oncology–Pathology, Radiumhemmet, Karolinska Hospital, S-171 76, Stockholm, Sweden

**Table 1** Patient characteristics

| Patient no | Sex[a] | Age (years) | Site | TNM[b] | Clinical stage | Clinical response[c] | $G_2$ phase fraction[d] |
|---|---|---|---|---|---|---|---|
| 1 | M | 50 | Larynx | T3N0M0 | III | PR | 2.31 |
| 2 | M | 50 | Oropharynx | T3N1M0 | III | CR | 2.73 |
| 3 | M | 72 | Oral cavity | T4N0M0 | IV | CR | 0.79 |
| 4 | M | 60 | Larynx | T2N0M0 | II | CR | 3.35 |
| 5 | M | 54 | Oral cavity | T2N2M0 | IV | PR | 2.53 |
| 6 | M | 63 | Oral cavity | T4N0M0 | IV | PR | 1.70 |
| 7 | M | 56 | Oropharynx | T4N1M0 | IV | CR | 2.12 |
| 8 | M | 70 | Oral cavity | T3N3M0 | IV | PR | 4.17 |
| 9 | F | 66 | Larynx | T1N1M0 | III | CR | 1.95 |
| 10 | M | 66 | Oral cavity | T4N3M0 | IV | PR | 1.68 |
| 11 | M | 63 | Oropharynx | T3N0M0 | III | CR | 1.17 |
| 12 | F | 57 | Hypopharynx | T2N0M0 | II | CR | 2.35 |
| 13 | M | 54 | Oral cavity | T3N0M0 | III | PR | 2.14 |
| 14 | F | 75 | Oropharynx | T3N0M0 | IV | NR | 1.17 |
| 15 | M | 83 | Oropharynx | T4N0M0 | IV | CR | 1.96 |
| 16 | F | 81 | Larynx | T3N0M0 | III | CR | 1.44 |
| 17 | M | 81 | Oropharynx | T3N0M0 | III | CR | 1.64 |
| 18 | F | 53 | Larynx | T2N1M0 | III | CR | 1.75 |
| 19 | F | 69 | Hypopharynx | T4N0M0 | IV | CR | 1.27 |
| 20 | M | 76 | Larynx | T2N0M0 | II | CR | 1.55 |
| 21 | M | 63 | Oropharynx | T4N2Mx | IV | NR | 1.35 |
| 22 | M | 64 | Larynx | T3N0M0 | III | CR | 1.74 |
| 23 | M | 49 | Oropharynx | T3N0M0 | III | CR | 3.09 |
| 24 | M | 61 | Oropharynx | T2N3M0 | IV | CR | 1.63 |
| 25 | F | 65 | Hypopharynx | T2N0M0 | II | CR | 0.75 |
| 26 | M | 85 | Larynx | T2N0M0 | II | CR | 1.00 |
| 27 | F | 75 | Larynx | T1N0M0 | I | CR | 0.96 |
| 28 | M | 47 | Larynx | T3N0M0 | III | NR | 0.80 |
| 29 | M | 66 | Larynx | T2N0M0 | II | CR | 0.83 |
| 30 | M | 72 | Larynx | T2N0M0 | II | CR | 1.71 |
| 31 | M | 72 | Oropharynx | T3N3M0 | IV | CR | 1.60 |
| 32 | M | 73 | Oral cavity | T2N0M0 | II | CR | 2.01 |
| 33 | M | 56 | Larynx | T2N0M0 | II | CR | 2.05 |
| 34 | M | 65 | Hypopharynx | T2N2M0 | IV | CR | 2.36 |
| 35 | M | 70 | Larynx | T2N0M0 | II | CR | 1.66 |
| 36 | F | 61 | Oropharynx | T4N0M0 | II | CR | 2.12 |
| 37 | F | 49 | Oral cavity | T2N0M0 | II | PR | 1.20 |
| 38 | F | 54 | Oropharynx | T2N1M0 | III | CR | 0.90 |
| 39 | F | 66 | Oral cavity | T3N0M0 | III | NR | 1.10 |
| 40 | F | 49 | Oral cavity | T2N0M0 | II | CR | 0.57 |
| 41 | M | 71 | Larynx | T2N0M0 | II | CR | 4.78 |
| 42 | M | 92 | Oral cavity | T4N3M0 | IV | CR | 2.38 |
| 43 | M | 62 | Larynx | T2N0M0 | II | CR | 2.74 |
| 44 | M | 49 | Hypopharynx | T2N0M0 | II | CR | 2.32 |
| 45 | M | 80 | Oral cavity | T4N2M0 | IV | NR | 1.09 |
| 46 | M | 50 | Oral cavity | T2N0M0 | II | CR | 1.89 |
| 47 | M | 55 | Oropharynx | T2N0M0 | II | CR | 2.02 |
| 48 | M | 41 | Oral cavity | T2N0M0 | II | CR | 2.22 |
| 49 | M | 71 | Hypopharynx | T1N0M0 | I | CR | 1.20 |

[a]M, male; F, female. [b]TNM, tumour node metastasis. [c]Type of clinical response after full-course radiotherapy with a final tumour absorbed dose of 64 Gy. CR, complete responders; PR, partial responders; NR, non-responders. [d]Mean fraction of cells passing to $G_2$ phase after X-ray irradiation, relative to background.

healthy controls were male. External RT was delivered with 4–8 MV X-rays in daily fractions of 2 Gy, five fractions per week, with a final tumour-absorbed dose of 64 Gy. Sampling of blood from the patients was approved by the medical ethical committee of the Karolinska Institute.

Our patient material covers patients with various types of squamous cell carcinoma of the head and neck, and Table 1 lists the site and stage distributions. The categories of clinical remission at the end of RT and/or in the following 2-month period are: complete response (CR), a complete disappearance of the tumour; partial response (PR), 50–99% decrease of tumour size (the product of two perpendicular diameters); no response (NR), 0–49% decrease in tumour size. Clinical information on type of remission was analysed in a blinded fashion. The code was then broken and clinical remission was compared with the in vitro findings.

### Cell culture, irradiation and flow cytometry

The PBL fraction along with other mononuclear cells was isolated from blood samples by differential centrifugation in lymphoprep (Nycomed, density 1.077, speed 750 *g* at 4°C for 20 min). The mononuclear preparation was purified of macrophages using the

*© Cancer Research Campaign 1998*

technique of iron absorption. The cells were then stimulated using a combination of 6 μg ml$^{-1}$ concanavalin A (Con A) and 25 ng ml$^{-1}$ phorbol 12-myristate 13-acetate (PMA). These drugs were used because of their combined effects in initiating two different pathways that participate in cell activation and cell cycle progression (Crabtree, 1989). Cells were incubated at 37°C in RPMI 1640 medium supplemented with 10% fetal calf serum, 1% Hepes buffer, L-glutamine (2 mM) and PEST (penicillin 2.4 IU ml$^{-1}$ and streptomycin 2.4 μg ml$^{-1}$). The initial concentration of cells in culture was 2.5–3.5 × 10$^{6}$ cells ml$^{-1}$. Twenty-four hours after mitogen stimulation the cells were irradiated (0, 2, 4 and 8 Gy) in culture medium at ambient room temperature (Siemens, 250 kV, 15 mA, 0.5-mm Cu-filter, SSD 50 cm, 0.81 Gy min$^{-1}$). After a further 48 h of incubation at 37°C, the cells were fixed.

For single-parameter DNA analysis, the cells were fixed in phosphate-buffered formalin (4% formaldehyde (w/w), 29 mM sodium dihydrogenphosphate × 2 $H_2O$, 47 mM disodium hydrogenphosphate × 2 $H_2O$, pH 7.0) at different times up to 72 h after mitogen stimulation. After a fixation time of about 16 h, the formalin was replaced by 95% ethanol and the samples were kept at 4°C. The cells were then incubated in distilled water, treated with subtilisin Carlsberg (0.1% Sigma protease XXIV, 0.1 M Tris, 0.07 M sodium chloride, pH 7.5) and stained by adding DAPI-SR101 solution (8 μM DAPI, 50 μM sulphorhodamine 101, 0.1 M Tris, 0.07 M sodium chloride, pH 7.5) (Castro et al, 1993). For two-parameter DNA–protein analysis, the cells were directly fixed in 95% ethanol and kept at 4°C. The staining was performed using DAPI-SR101 solution (8 μM DAPI, 50 μM sulphorhodamine 101, 0.1 M Tris, 0.07 M sodium chloride, pH 7.5) as described before (Heiden et al, 1990). All flow analyses were performed using a PAS II flow cytometer (Partec, Münster, Germany). Up to 50 000 cells were measured per histogram. The protein analysis was internally standardized using fluorescent beads (6.4 μm Polychromatic beads; Polysciences, Warrington, CA, USA). During a 10-month period, the analysis of mouse Ehrlich ascites tumour cells fixed in 95% ethanol was repeated using the beads as an internal reference. The mean ± s.d. of the protein values in $G_1$ cells was 109.8 ± 8.4 ($n$ = 10), which demonstrates a good reproducibility of the protein analysis. Gating analysis and calculations of protein median values were performed with the help of the PAS II software. For calculation of cell cycle composition, the Multicycle program (Phoenix Flow Systems, San Diego, CA, USA) was used.

We found that, 24 h after the start of mitogen stimulation, cells had entered the $G_1$ phase (Figure 1) and, 48 h later, approximately 25% of the cells were in S + $G_2$ (Figures 1 and 2), without changing the total cell number (data not shown). Hence, very few, if any, cells had entered a second cell cycle. Samples with < 10% S + $G_2$ in unirradiated cells 72 h after start of mitogen stimulation were excluded from further evaluation.

### Definition of variables used

$G_1(D)$, $S(D)$ and $G_2(D)$ represent the proportion of $G_1$, S- and $G_2$ phase cells, respectively, after exposure to dose $D$. $FG_2(D) = G_2(D)/[G_2(D) + S(D)]$ determines the fraction of cells passing to $G_2$ after exposure to dose $D$, as related to the population of proliferating cells. For each patient and healthy donor, the response variables analysed were $S(D)/S(0)$ and $FG_2(D)/FG_2(0)$. These relative S- and $G_2$-phase fractions describe the response to dose $D$ as related to background.

**Figure 1** Protein analysis of gated $G_{0/1}$ lymphocytes at different times after mitogenic stimulation. The histograms show a serial analysis of cells from one healthy donor. In addition, the fractions of S + $G_2$/M cells are shown. The histogram at the bottom shows the correlated DNA–protein analysis at 72 h. The rectangle labels the $G_{0/1}$ gating window. In all analyses, internal standard beads were measured in DNA channel number 0 and the protein channel number 64, which is marked by S. The median protein values of the $G_{0/1}$ cells were calculated in each histogram and the means ± s.d. of these protein values were calculated for several donors (triangle, mean; solid line, s.d.). 0 h, 81 ± 7 ($n$ = 3); 24 h, 117 ± 42 ($n$ = 16); 72 h, 166 ± 55 ($n$ = 4). The 24-h mean protein $G_{0/1}$ value does not differ significantly from either the 0-h value ($P$ = 0.16) or the 72-h value ($P$ = 0.06). The 0-h value differs significantly from the 72-h value ($P$ = 0.048)

### Statistical analysis

The data sets, based on the three different clinical responder groups and the two healthy donor groups, were analysed by repeated measurement ANOVA. Tests of differences between groups, differences in dose dependence between individuals and between groups (i.e. presence of dose–group interaction) were carried out. Pairwise comparisons were also carried out by

© Cancer Research Campaign 1998

**Figure 2** Percentages of peripheral blood lymphocytes from two individuals in the various cell cycle phases at different times after stimulation with PMA and Con A. ●, $G_{0/1}$; ▲, S; ■, $G_2$/M. The inset shows two representative DNA histograms of lymphocytes at 0 (top) and 72 (bottom) h after stimulation. x-axis, relative DNA content per cell; y-axis, relative cell number

**Figure 3** (**A**) Dose–response curves for ten healthy donors expressed as the fraction of cells passing to S-phase, relative to background. Donors 1–5 represent split samples and 6–10 resampled donors. (**B**) Dose–response curves for ten healthy donors expressed as the fraction of cells passing to $G_2$ phase, relative to background. Donors 1–5 represents split samples, and 6–10 resampled donors defining appropriate contrasts. In the analysis of donor and assay variability, in which each donor had five determinations, tests exploring inter-donor variability, dose dependence and dose–donor interaction were performed. Comparisons of intra-assay and intra-donor variation have been carried out comparing the residual variance for the two groups of healthy donors.

The data variables, defined as the logarithm of the ratio between the cell cycle phase values of the exposed and the control samples, i.e. the natural logarithm of $G_1(D)/G_1(0)$, $S(D)/S(0)$ and $FG_2(D)/FG_2(0)$, were also subjected to two different kinds of multivariate analyses. The first method used was a classificatory discriminant analysis (Dillion and Goldstein, 1984). This analysis was used to classify observations into two or more known groups (five groups in the present material) on the basis of one or more quantitative variables. The second method used was a cluster analysis (Everitt, 1980), searching for five distinct groups of individuals based on the nine variables (i.e. different cell cycle stages and radiation doses) for each subject; however this was in contrast to the discriminant analysis, as the categorization was not used.

### RESULTS

### Analysis of healthy donor: intra-assay, intra- and inter-donor variability

Analysis of intra-donor and intra-assay variability was carried out using blood samples from ten normal donors. Fresh blood samples from five donors were split into five aliquots and processed for the analysis of intra-assay variability. The remaining five donors were resampled at five different times for the analysis of intra-donor variability. Figures 3A and B show the dose dependence of the two end points for each individual. The dose–response effect is highly significant for both end points ($P < 0.001$). The analysis shows a significant overall variation between donors for relative S-phase fraction ($P < 0.001$) but not for relative $G_2$ phase fraction ($P = 0.93$). However, for the S-phase fraction, this inter-donor variation is almost totally accounted for by a single person (donor 3). The dose–donor interaction has also been tested for both end points and was found to be significant for relative $G_2$ phase fraction ($P < 0.001$) but not for relative S-phase fraction. Donor 3 deviates with no increase in response from 4 to 8 Gy and, when this donor is excluded, the effect on relative $G_2$ phase fraction for dose–donor interaction is only marginal. No significant difference in variability between the intra-assay (donors 1–5) and the intra-donor (donors 6–10) samples could be detected. The results obtained in the intra-assay and intra-donor test showed, for the two analyses, both high and low degrees of reproducibility at the individual level. However, the donor heteroscedasticity in the group of healthy controls is clearly dominated by the intra-assay variability.

### Analysis of head and neck cancer patients and healthy donors

The clinical information, including the clinical response to RT, is summarized in Table 1 together with the relative $G_2$ phase data. Thirty-seven patients achieved a CR, seven patients a PR and the

© Cancer Research Campaign 1998

**Figure 4** (**A**) Dose–response curves for 49 head and neck cancer patients with different remission after conventional radiotherapy and 15 healthy donors expressed as mean ± s.e.m. fraction of S-phase cells, relative to background, in peripheral blood lymphocytes at 72 h after mitogen stimulation and 48 h after irradiation with 2, 4 or 8 Gy. ●, CR ($n$ = 37); ▲, PR ($n$ = 7); ■, NR ($n$ = 5); ○, H1 ($n$ = 15). (**B**) Dose–response curves for 49 head and neck cancer patients with different remission after conventional radiotherapy and 15 healthy donors expressed as mean ± s.e.m. fraction of cells passing to $G_2$ phase, relative to background. Cells were assayed as above. ●, CR ($n$ = 37); ▲, PR ($n$ = 7); ■, NR ($n$ = 5); ○, H1 ($n$ = 15)

**Table 2** Significance for pairwise comparisons between groups[a]

| | CR | PR | NR | H1 |
|---|---|---|---|---|
| CR | | 0.019 | 0.016 | < 0.001 |
| PR | 0.19 | | < 0.001 | < 0.001 |
| NR | 0.034 | 0.009 | | 0.55 |
| H1 | 0.056 | 0.016 | 0.4 | |

[a]The values above the diagonal correspond to the S-phase end point and the values under the diagonal to the $G_2$ phase end point.

remaining five patients belonged to the group of NR. Figure 4A shows the mean ± s.e.m. of the relative S-phase fraction for each group. Statistical analysis shows that there is a difference in the level of response between the four groups (i.e. the three patient groups and the H1 group; overall test $P < 0.001$). There is also a clear overall dose–response ($P < 0.001$) but no significant dose–group interaction ($P = 0.69$). Figure 4B shows the corresponding means ± s.e.m. of the relative $G_2$ phase fraction for each

**Table 3** Result of discriminant analysis: observed type by predicted type[a]

| | Predicted type | | | | | |
|---|---|---|---|---|---|---|
| **Group** | **CR** | **PR** | **NR** | **H1** | **H2** | **Total** |
| CR | 17 | 5 | 6 | 5 | 3 | 36 |
| PR | 0 | 6 | 0 | 0 | 0 | 6 |
| NR | 0 | 0 | 5 | 0 | 0 | 5 |
| H1 | 2 | 0 | 0 | 13 | 0 | 15 |
| H2 | 0 | 0 | 0 | 0 | 10 | 10 |
| Total | 19 | 11 | 11 | 18 | 13 | 72 |

[a]Two patients were not admissible for analysis because of zero value in S-phase for one CR (8 Gy) and for one PR (4 Gy).

**Table 4** Result of cluster analysis: observed type by cluster type[a]

| | Cluster type | | | | | |
|---|---|---|---|---|---|---|
| **Group** | **1** | **2** | **3** | **4** | **5** | **Total** |
| CR | 2 | 9 | 6 | 9 | 10 | 36 |
| PR | 1 | 4 | 0 | 0 | 1 | 6 |
| NR | 0 | 1 | 3 | 1 | 0 | 5 |
| H1 | 0 | 0 | 4 | 11 | 0 | 15 |
| H2 | 0 | 0 | 0 | 10 | 0 | 10 |
| Total | 3 | 14 | 13 | 31 | 11 | 72 |

[a]Two patients were not admissible for analysis because of zero value in S-phase for one CR (8 Gy) and for one PR (4 Gy).

group. Analysis of this end point shows a difference between the four groups (overall test $P = 0.016$) and a significant dose–response ($P = 0.034$) but no significant dose–group interaction ($P = 0.43$). Table 2 displays the significance for the pairwise comparison between the different groups. The values above the diagonal correspond to the $P$-values of the S-phase comparisons and show that CR differed significantly from both H1, NR and PR. As can be seen in Figure 4A, the relative S-phase values in the CR group were lower than in the H1 and NR and higher than in the PR group. Figure 4B shows that the relative $G_2$ fractions in the CR group were higher than in the NR group and that the values of the PR cases were higher than in both H1 and NR. Table 2 shows that these differences were statistically significant. None of the end points showed any statistically significant correlation to tumour site, stage, sex or age ($P > 0.05$).

### Multivariate statistical analysis

Table 3 shows the results of a discriminant analysis. There is good agreement between the predicted category and the observed category for H1, H2, PR and NR, but not for CR. There is no overlap between the two healthy donor groups (H1 and H2). For PR and NR, 100% of the patients were correctly classified. The second type of multivariate method used was a cluster analysis. Table 4 shows how the five categories of subjects fall into the five clusters found by the algorithm. Again CR seems to be difficult to categorize falling with about equal proportions in clusters 2–5. Most of the healthy donors fall into cluster 4, showing that the subjects in the two healthy groups are 'close' to each other; most of the PR and NR subjects belong to clusters 2 and 3.

© Cancer Research Campaign 1998

### DISCUSSION

It is of interest to determine whether one can predict the radiosensitivity of tumour tissues based on evaluation of PBLs. In the present study, we addressed this question for patients with head and neck cancer scheduled for curative RT. The in vitro end point was the fraction of PBLs passing to S and $G_2$ after irradiation. We used early response as an in vivo indicator of tumour radiosensitivity. Jaulerry et al (1995) showed that tumour regression during and at completion of external RT (early response) is an independent predictive factor of local control in head and neck. When comparing accumulation of cells in S and $G_2$, NR cases differed from CR and PR cases in that they displayed higher S and lower $G_2$ accumulation (Figures 4A and B). One may hypothesize that these results reflect a difference in a radiation-sensitive cell cycle checkpoint. As the cells were assayed 48 h after irradiation, the exact position of such a checkpoint may be difficult to pinpoint. NR cases may have a radiation-sensitive checkpoint in $G_1$ or S, which is more effective than for CR and PR cases, whose PBLs progress more efficiently to $G_2$ before they arrest. However, we did not find it possible to predict NR for an individual patient, because of variability between the groups. The PBLs from healthy donors (H1) showed a response similar to that of the NR patients and a significantly different cell cycle progression compared with PR. The comparison with CR approached statistical significance for the S-phase end point and reached significance for the $G_2$ phase end point. Furthermore, discriminant and cluster analysis show that grouping is possible. The discriminant analysis presumes that subject categorization is known. Cluster analysis is on the other hand an 'internal' method, in that the individuals are not classified by any criteria other than the variables used in the analysis. Using this latter approach, it was possible to categorize most of the PR and NR subjects into clusters. The evidence provided by this analysis does not need validation to the same extent as the discriminant analysis as the a priori categorization was not used. The result of this analysis underlines that there is discriminating power in the measurements.

We also analysed PBLs from healthy donors to study the intra-assay and intra-donor variability. Most of the individuals in the two healthy donor groups (H1 and H2) were grouped into the same clusters using multivariate analysis, which differs from the dose–response seen when comparing the two groups (data not shown). However, the H2 group was assayed almost 1 year after the H1 group, and this temporal difference might have had an influence on our results. The overall variation in dose dependence between healthy donors was significant in $G_2$ phase accumulation, but as the intra-assay variability dominates over the variability seen in the resampled donors, the interpretation is limited and not yet fully understood.

Individuals vary in their sensitivity to ionizing radiation. Defined disorders with radiosensitive phenotypes, such as the ataxia–telangiectasia (AT) syndrome, show that genetic factors can strongly determine the radiosensitivity of humans (Thacker, 1994). In addition, studies have revealed that cells from AT heterozygotes show increased radiosensitivity (Cole et al, 1988; Weeks et al, 1991; West et al, 1995). There are ongoing efforts to evaluate a possible role of AT heterozygosity for over-reaction to RT, particularly in breast cancer patients (Weeks et al, 1991; West et al, 1995). From these studies, it can be observed that in vitro sensitivity of normal cells from some cancer patients may fall into a similar range, as AT heterozygotes do. Interestingly, recent studies have reported changes in cell cycle kinetics after irradiation of AT heterozygote lymphoblastoid cells that are similar to those presented here (Lavin et al, 1992, 1994; Naeim et al, 1994).

Only a few studies have suggested the existence of a relationship between normal tissue and tumour radiosensitivity. Dahlberg et al (1993) reported a significant correlation between the radiosensitivities of sarcoma cells and fibroblasts derived from the same patient. Whether this correlation had a bearing on clinical tumour response is not known. Furthermore, a recent study suggests the association between tumour control and acute mucosal reaction after RT (Geara et al, 1996). If genetic differences are abundant that influence intrinsic radiosensitivity of both normal tissue and tumour response to RT, the measurements of such differences could guide us to a more appropriate dosing of RT. Usually the limiting factor in RT treatment is the maximum-tolerated dose of normal tissue. The doses used in conventional RT are relatively low to avoid problems in a radiosensitive subgroup of patients. This may result in underdosage in most of the patients. It has been estimated that identification of the most radiosensitive individuals would allow the use of higher radiation doses in the treatment of the remaining patients, without increasing side-effects (Norman et al, 1988). Therefore, it would also be of great interest to evaluate the relationship between the cell cycle kinetics of PBLs after in vitro irradiation and clinical normal tissue reponse to RT.

Lymphocytes are known to undergo apoptosis after irradiation (Sellins and Cohen, 1987). The DNA histograms in the present study show events in the sub-$G_1$ region in the stimulated (and irradiated) samples, as shown in Figures 1 and 2. This sign of DNA loss or nuclear fragmentation indicates cell death. Thus, it is possible that our observations of differences in cell cycle distributions are influenced by apoptosis that could occur with cell cycle phase specificity. However, substantial cell cycle progression occurs after stimulation of PBLs, and it is our impression that the present results mainly reflect differences in cell cycle kinetics.

In conclusion, although the present assay showed a discriminating power between the clinical response groups, the results of the test did not permit prediction of individual outcome of RT with satisfying accuracy.

### ACKNOWLEDGEMENTS

We thank Dr Juan Castro, Ms Christina Bohman and Ms Britt-Mari Graf for help with the DNA flow cytometry measurements. This work was supported by grants from the Gustav V Jubilee Found, the Cancer Society in Stockholm (95:120) and the Swedish Radiation Protection Institute (620:92).

### REFERENCES

Bentzen SM (1997) Potential clinical impact of normal-tissue intrinsic radiosensitivity testing. *Radiother Oncol* **43**: 121–131

Brock WA, Baker FL and Peters LJ (1989) Radiosensitivity of human head and neck squamous cell carcinomas in primary culture and its potential as a predictive assay of tumour radiocurability. *Int J Radiat Biol* **56**: 751–760

Castro J, Heiden T, Wang N and Tribukait B (1993) Preparation of cell nuclei from fresh tissues for high-quality DNA flow cytometry. *Cytometry* **14**: 793–804

Cole J, Arlett CF, Green MHL, Harcourt SA, Priestley A, Henderson L, Cole H, James SE and Richmond F (1988) Comparative human cellular radiosensitivity. II. The survival following gamma-irradiation of unstimulated ($G_0$) T-lymphocytes, T-lymphocyte lines, lymphoblastoid cell lines and fibroblasts from normal donors, from ataxia-telangiectasia patients and from ataxia-telangiectasia heterozygotes. *Int J Radiat Biol* **54**: 929–943

© Cancer Research Campaign 1998

Crabtree GR (1989) Contingent genetic regulatory events in T lymphocyte activation. *Science* **243**: 355–361

Dahlberg WK, Little JB, Fletcher JA, Suit HD and Okunieff P (1993) Radiosensitivity *in vitro* of human soft tissue sarcoma cell lines and skin fibroblasts derived from the same patients. *Int J Radiat Biol* **63**: 191–198

Dillion W and Goldstein M (1984) *Multivariate Analysis: Methods and Applications*. John Wiley & Sons: New York

Elyan SAG, West CML, Roberts SA and Hunter RD (1993) Use of low-dose rate irradiation to measure the intrinsic radiosensitivity of human T-lymphocytes. *Int J Radiat Biol* **64**: 375–383

Everitt BS (1980) *Cluster Analysis*, 2nd edn. Heineman Educational Books: London

Geara FB, Peters LJ, Ang KK, Wike JL, Sivon SS, Guttenberger R, Callender DL, Malaise EP and Brock WA (1992) Intrinsic radiosensitivity of normal human fibroblasts and lymphocytes after high- and low-dose-rate irradiation. *Cancer Res* **52**: 6348–6352

Geara FB, Peters LJ, Ang KK, Wike JL and Brock WA (1993) Prospective comparison of in vitro normal cell radiosensitivity and normal tissue reactions in radiotherapy patients. *Int J Radiat Oncol Biol Phys* **27**: 1173–1179

Geara FB, Peters LJ, Ang KK, Garden AS, Tucker SL, Levy LB and Brown BW (1996) Comparison between normal tissue reactions and local tumor control in head and neck cancer patients treated by definitive radiotherapy. *Int J Radiat Oncol Biol Phys* **35**: 455–462

Green MH, Arlett CF, Cole J, Harcourt SA, Priestley A, Waugh APW, Stephens G, Beare DM, Brown NAP and Shun-Shin GA (1991) Comparative human cellular radiosensitivity. III. γ-Radiation survival of cultured skin fibroblasts and resting T-lymphocytes from the peripheral blood of the same individual. *Int J Radiat Biol* **59**: 749–765

Heiden T, Göhde W and Tribukait B (1990) Two-wavelength mercury arc lamp excitation for flow cytometric DNA-protein analyses. *Anticancer Res* **10**: 1555–1562

Jaulerry C, Dubray B, Brunin F, Rodriguez J, Point D, Blaszka B, Asselain B, Mosseri V, Brugere J and Cosset J-M (1995) Prognostic value of tumor regression during radiotherapy for head and neck cancer: a prospective study. *Int J Radiat Oncol Biol Phys* **33**: 271–279

Kushiro JI, Nakamura N, Kyoizumi S, Nishiki M, Dohi K and Akiyama M (1990) Absence of correlations between radiosensitivities of human T-lymphocytes in $G_0$ and skin fibroblasts in log phase. *Radiat Res* **122**: 326–332

Lavin MF, Poidevin PL and Bates P (1992) Enhanced levels of radiation-induced $G_2$ phase delay in ataxia telangiectasia heterozygotes. *Cancer Genet Cytogenet* **60**: 183–187

Lavin MF, Bennet I, Ramsay J, Gardiner RA, Seymor GJ, Farrell A and Walsh M (1994) Identification of a potentially radiosensitive subgroup among patients with breast cancer. *J Natl Cancer Inst* **86**: 1627–1634

Naeim A, Repinski C, Huo Y, Hong J-H, Chessa L, Naeim F and Gatti RA (1994) Ataxia–telangiectasia: flow cytometric cell-cycle analysis of lymphoblastoid cell lines in $G_2$/M before and after γ-irradiation. *Methods Pathol* **7**: 587–592

Nakamura N, Sposto R, Kushiro JI and Akiyama M (1991) Is interindividual variation of cellular radiosensitivity real or artifactual? *Radiat Res* **125**: 326–330

Norman A, Kagan AR and Chan SL (1988) The importance of genetics for the optimization of radiation therapy. *Am J Clin Oncol* **11**: 84–88

Ramsay J and Birrell G (1995) Normal tissue radiosensitivity in breast cancer patients. *Int J Radiat Oncol Biol Phys* **31**: 339–344

Sellins KS and Cohen JJ (1987) Gene induction by γ-irradiation leads to DNA fragmentation in lymphocytes. *J Immunol* **139**: 3199–3206

Thacker J (1994) Cellular radiosensitivity in ataxia–telangiectasia. *Int J Radiat Biol* **66**: S87–S96

Weeks DE, Paterson MC, Lange K, Andrais B, Davis RC, Yoder F and Gatti RA (1991) Assessment of chronic γ radiosensitivity as an *in vitro* assay for heterozygote identification of ataxia–telangiectasia. *Radiat Res* **128**: 90–99

West CML (1994) Predictive assays in radiation therapy. *Adv Radiat Biol* **18**: 149–180

West CML, Davidson SE, Hendry JH and Hunter RD (1991) Prediction of cervical carcinoma response to radiotherapy. *Lancet* **338**: 818

West CML, Elyan SAG, Berry P, Cowan R and Scott D (1995) A comparison of the radiosensitivity of lymphocytes from normal donors, cancer patients, individuals with ataxia–telangiectasia (A-T) and A-T heterozygotes. *Int J Radiat Biol* **68**: 197–203

© Cancer Research Campaign 1998